# Investigating GERMs: how genotype, environment, and rhizosphere microbiome interactions underlie heat response in maize and sorghum

**DOI:** 10.1111/nph.71297

**Published:** 2026-05-26

**Authors:** Nate Korth, Isabella Borrero, Katelyn Rumley, Alex L. Woodley, Mallory J. Choudoir, Joseph L. Gage

**Affiliations:** ^1^ Department of Crop and Soil Sciences North Carolina State University Raleigh NC 27695 USA; ^2^ NC Plant Sciences Initiative North Carolina State University Raleigh NC 27606 USA; ^3^ Department of Plant and Microbial Biology North Carolina State University Raleigh NC 27695 USA

**Keywords:** abiotic stress tolerance, cross‐kingdom regulation, d‐amino acid metabolism, heat tolerance, metatranscriptomics, microbiome, plant resilience, rhizosphere biology

## Abstract

- Plant responses to heat stress emerge from interactions among host genotype, environment, and the rhizosphere microbiome, yet most studies examine these components in isolation. We applied the Genotype × Environment × Rhizosphere Microbiomes (GERMs) framework to test how host–microbe coordination contributes to heat tolerance in cereal crops *Zea mays* and *Sorghum bicolor*.
- We analyzed maize and sorghum grown under optimal and heat‐stressed conditions across contrasting soil treatments using integrated plant–microbial metatranscriptomics. Host and microbial gene expression profiles were jointly analyzed alongside microbiome composition and plant phenotypes and compared with amplicon‐based profiling.
- Metatranscriptomics captured microbial community structure comparable to amplicon sequencing while providing enhanced functional and taxonomic resolution. Host genotype and temperature jointly shaped microbial functional profiles. Conserved plant orthologs across maize and sorghum were linked to microbial pathways, specifically microbial d‐amino acid metabolism was associated with plant heat tolerance.
- These findings indicate the rhizosphere microbiome actively participates in plant heat stress responses through coordinated transcriptional interactions with the host. Integrating host and microbial transcriptomes reveals mechanistic insights into plant adaptation and establishes a framework for dissecting plant–microbiome interactions under environmental stress.

## Graphical abstract

Three genotypes – a heat‐resistant maize (*Zea mays*), a heat‐susceptible maize, and a sorghum (*Sorghum bicolor*) variety – were grown to the V4 stage in growth chambers under optimal conditions or subjected to heat stress. Plants were grown in soil containing a complex microbial community, or in the same soil with a depleted microbiome. Total RNA from roots and root‐associated microbes was sequenced, along with the 16S rRNA amplicon from both DNA and RNA. Plants were assigned a qualitative heat stress score based on size and leaf senescence (bottom right), as well as quantitative metrics, including biomass and root architecture.

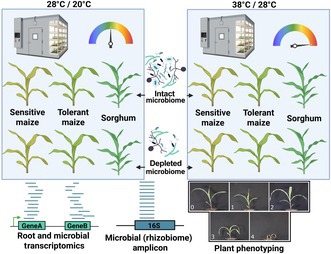

## Introduction

Genotype‐by‐environment (G × E) interactions are a central area of research for the future of sustainable agriculture (ComStock & Moll, 1963; Steiner *et al*., 2021). While it is well established that an organism's phenotype is shaped by its genotype, the environment, and their interaction, most GxE models do not account for the host‐associated microbiome. The recognition of the host and its associated microbes as a single functional unit – a holobiont – has fundamentally reshaped our understanding of biology (Vandenkoornhuyse *et al*., 2015; Addison *et al*., 2024). Recent studies have demonstrated that G × E can significantly impact mutualism‐related traits in legumes, among many other phenotypes (Vaidya & Stinchcombe, 2020; Plett *et al*., 2021). Given the crucial role of the host microbiome in fitness, we echo Oyserman *et al*. (2021) and propose that microbiome information be incorporated into G × E models as a distinct term. The Genotype, Environment, Rhizosphere, and Microbiome (GERM) interactions framework allows us to dissect these complex relationships, providing a deeper understanding of the effects of each component and their interactions on host phenotypes. In this paper, we apply this framework to identify how the rhizosphere microbiome contributes to plant adaptation to heat stress and underlies G × E mechanisms.

The GERMs framework is particularly useful for addressing the challenges posed by climate change, as rising global temperatures present an immense threat to agriculture (Hultgren *et al*., 2025). 2024 was the warmest year on record, with global average temperatures 1.55°C above the preindustrial average (Lindsey *et al*., 2024). High temperatures early in the growing season can increase crop growth rates, but also have damaging effects on plant maturation and grain fill (Delouche & Baskin, 2021). Bacterial and fungal members of the rhizosphere microbiome are known to increase thermotolerance in multiple plant species, likely by preactivating heat‐shock transcription factors and upregulating plant secondary metabolites (Abd El‐Daim *et al*., 2014; Maitra *et al*., 2021). Many plants, including cereal crops such as *Zea mays* L. (maize) and *Sorghum bicolor* (sorghum), recruit specific rhizosphere microbes under heat and other abiotic stress through the production of specific root exudates (Chai & Schachtman, 2022; Tiziani *et al*., 2022).

Cereal crops, which account for more than half of the calories consumed by humans world‐wide, are threatened by climate change (Awika, 2011). More than 1 billion tons of maize are produced world‐wide each year, making it the world's most‐produced grain and an extremely important crop for global food and fuel production (Food and Agriculture Organization of the United Nations, 2020). Thus, minor improvements to the sustainability of modern maize can have a massive impact. While sorghum is globally important, ranking as the fifth most widely grown cereal, it is also a close relative of maize and shares many orthologous genes (Swigoňová *et al*., 2004). Sorghum, through its human‐aided adaptation across nearly all of continental Africa, also exhibits tolerance to many abiotic stresses, including heat and drought (Chopra *et al*., 2017; Abreha *et al*., 2021). While maize and sorghum may share some mechanisms for resisting abiotic stress conserved across evolutionary time, mechanisms of resilience unique to sorghum could be incorporated into maize through gene editing, and insights into possible differences in sorghum‐microbe interactions vs maize‐microbe interactions could also prove useful. While genetic mechanisms are often conserved, mechanisms of microbiome recruitment and signaling based on microbial function remain obscured by methods that focus solely on microbial community composition.

While the field has made strides in understanding plant–microbe interactions under climate stress, much of the existing literature focuses on a single crop species and a single plant genotype or variety within that species. The few studies examining the microbiomes of multiple plant species or genotypes under abiotic stress conditions rely on microbiome amplicon sequencing (Hao *et al*., 2020; Oyserman *et al*., 2021; Brisson *et al*., 2022). Research on the human microbiome suggests that while the microbial composition of a given body site varies significantly between individuals, the microbial functional profile, as quantified by transcriptomics or proteomics, remains remarkably conserved (Huttenhower *et al*., 2012). While there is a lack of overlapping compositional and functional datasets from the plant rhizosphere microbiome, compositional data across multiple environments show high variation between field sites (environments) (Walters *et al*., 2018). Despite strong environmental drivers of rhizosphere composition, inferred functional enrichment showed far less variation in a phenomenon similar to that observed in humans (Schultz *et al*., 2023). To better understand the functional relationships among maize, the environment, and the plant‐associated microbiome, we employed a systems‐level metatranscriptomic approach. This method derives functional information from multiple kingdoms of life from a single RNA extraction and metatranscriptomic sequencing. We designed a bioinformatic workflow to separate mRNA from plants, bacteria, and fungi and to explore mechanisms underlying genetically controlled maize microbiome‐associated phenotypes.

In this study, we investigate how plant and rhizosphere microbial gene expression jointly respond to temperature stress and influence plant performance. By integrating transcriptomic data from both host and microbes into differential correlation and machine learning analyses, we identify plant genes and microbial pathways associated with heat–stress resilience. Specifically, we identify microbial d‐amino acid (DAA) metabolism as a key microbial function linked to plant fitness under stress. Our analysis supports a model in which the enrichment of this function is plant‐mediated, diverges from microbial stress responses in bulk soil, and is broadly distributed across bacterial phylogeny. We demonstrate that this systems‐level approach is a strategy for generating hypotheses about coordinated molecular mechanisms underlying plant resilience to abiotic stress.

## Materials and Methods

### Soil preparation

Field soil was collected from North Carolina State University's Lower Coastal Plain Research Station in Kingston, NC (35.378305, −77.559656) from fields with maize and wheat rotations, with the most recent rotation being maize, and kept in isolation for 60 d. The field soil is classified as Goldsboro loamy sand with a mean particle diameter (*D*_50_) of 0.16 mm and a plasticity index of 8. Complete soil chemistry is available in Supporting Information Table S1. A portion of the soil was then subjected to two rounds of autoclaving at 121°C for 60 min. The potting media was formulated with 60% field soil (autoclaved or unautoclaved), 30% sterilized calcined clay (Turface™), and 10% sterile perlite (Miracle‐Gro®). The soil was mixed in 6 l batches, including 30 ml of multicote (14–14–16) nutrient solution, and added to 4‐inch pots. Each pot was topped with a 1 cm layer of the autoclaved soil to reduce weed growth. Pots were then watered with RO water for 1 wk before the start of the experiment to remove any weed seedlings that persisted.

### Genotype selection

Two tropical, inbred *Zea mays* L. (maize) genotypes, a heat‐tolerant genotype, CML52 (PI 595561), and a heat‐susceptible genotype, CML103 (PI 690319), were obtained from a collection of the maize 282 panel grown to increase seed in Puerto Vallarta, Mexico, in 2022–2023 (McNellie *et al*., 2018; Gao *et al*., 2019). A moderately tolerant genotype of *Sorghum bicolor* (L.) Moench (sorghum), SAP‐166 (PI 597964), was obtained from the Southern Regional Plant Introduction Station (SRPIS) in Griffin, GA, in 2022 (USDA Agricultural Research, 2023). The inclusion of sorghum alongside maize was meant to provide a sample set that spanned a wide evolutionary distance without requiring a vast amount of genotypes. This design allows us to isolate core functional responses conserved across evolutionary time. To ensure a fair comparison between species, our analysis focused on orthologous genes in maize and sorghum following established methods in comparative transcriptomics (Vercruysse *et al*., 2020) (see ‘Bioinformatic Workflow – metatranscriptome’ section).

### Experimental design and growth chamber conditions

Each genotype was replicated four times in field and autoclaved soil at two temperature conditions. Autoclaved soils served as a microbiome‐depleted condition. Bulk soil in pots without plants at both temperatures were included in triplicate as controls. To address potential colonization by growth chamber environmental organisms and ensure the treatment effectively reduced microbial functionality, we examined metatranscriptomic diversity between samples with autoclaved and field soil. We determined autoclaved soil has lower functional alpha diversity and hereafter refer to it as a ‘depleted microbiome’ treatment (Fig. S1). To minimize differential colonization within treatments, pots were filled from a single homogenized batch of field or autoclaved soil media in a randomized complete block design across two growth chambers: one at normal growth conditions (28°C : 20°C, day : night) and the other under heat stress conditions (38°C : 28°C, day : night). The light intensity of both growth chambers was set to 320 μmol (s · m^2^)^−1^ (Zhang *et al*., 2022).

### Seedling germination

Seeds were sterilized by soaking in a 10% sodium hypochlorite solution with one drop of Tween® 20 on a rotating mixer for 20 min. Seeds were then triple‐rinsed with sterile H_2_O before soaking in 70% EtOH for 2 min. To finish, seeds were triple‐rinsed with sterile H_2_O. Filter paper was wet and layered on top of plastic wrap and wax paper. Seeds were then all placed one inch apart, two inches below the top of the filter paper with the germ pointed downward. A second piece of damp filter paper and wax paper were layered on top, and all five layers were then loosely rolled and placed into a large beaker with water, ensuring that seeds were not submerged. The beaker was then placed in a growth chamber set to 28°C : 20°C. After 2 d, the rolls were checked for germination and transplanted if germination had occurred. Pots were watered to saturation at transplanting and then as needed until sampling.

### Plant sampling for RNA and DNA extractions

The day of sampling was decided based on when the majority of the plants reached V3–V4 on the plant growth stage scale (Xiong *et al*., 2021). The morning of sampling, plant growth stage was noted, a photo of each pot was quickly taken, and plants were rated on a 0–4 categorical scale based on plant size and leaf senescence, where zero means the plant showed no sign of heat stress and four means that the plant showed extreme stress and was withered (Graphical Abstract).

Roots were sampled in sets of four plants, with two people working simultaneously. To remove plants, pots were gently squeezed to loosen soil and inverted onto trays. Excess soil was manually massaged from the roots, which were then gently shaken to remove loose particles. Large particulates were removed, and shoots were clipped at the base and set aside for biomass measurements.

Cleaned roots were sonicated to loosen adhered soil, microbes, and the outer layer of plant cells, yielding a mixture of microbial and plant nucleic acids in each sample. Roots were immediately fully submerged into prechilled 50 ml tubes containing 25 ml of 1× phosphate‐buffered saline (PBS; pH 7.4). Tubes were inverted five times, sonicated for 20 s, inverted, and sonicated for an additional 20 s, followed by five more inversions. Roots were then removed, leaving soil in suspension. Samples were centrifuged at 2500 ***g*** for 1 min at 4°C, and the supernatant was discarded. Pellets were flash‐frozen in liquid nitrogen and stored at −80°C until nucleic acid extraction.

### Plant phenotyping

In addition to the categorical heat stress score, biomass from cleaned roots and shoots was collected. Roots were then given an additional wash and scanned using a WinRhizo root scanner (Regent Instruments Inc., Québec, QC, Canada) equipped with the WinRhizo Pro software (v.2022b). Roots were carefully spread in a water‐filled, transparent tray to minimize overlap, then scanned at high resolution. Images were analyzed in WinRhizo/XLRhizo using the manufacturer's default calibration and classification settings to quantify root system architecture traits. A range of root phenotypes were extracted, including total root length, average diameter, surface area, volume, and topological parameters (e.g. tips, forks, crossings). The complete list of phenotypes measured, along with their corresponding values, is included in Table S2 (Metadata).

### DNA and RNA extractions

RNA and DNA were extracted from the frozen soil samples using the Qiagen PowerSoil DNA Kit (12866‐25) and the RNeasy PowerSoil Total RNA Kit (12867‐25), respectively.

### Amplicon sequencing (DNA)

We surveyed 16S rRNA genes from DNA and RNA to profile whole communities (active and dormant taxa) and active fraction, respectively. Extracted DNA was sent to the North Carolina State University Genomic Science Laboratory for Illumina 16S/ITS Amplicon library preparation and sequencing with 16S primers 322F‐A (ACGGHCCARACTCCTACGGAA) and 796R (CTACCMGGGTATCTAATCCKG) designed to amplify microbial 16S from plant tissue selectively (Chen *et al*., 2022). Genus‐level total read counts from the 16S amplicon sequencing are available in Dataset S1.

### Amplicon sequencing (RNA)

To create a cDNA library, remaining DNA was removed from RNA samples using the TURBO DNA‐free Kit (#AM1907; Invitrogen; Thermo‐Fisher Scientific; Waltham, MA, USA) DNase Kit. DNA‐free RNA samples were then reverse‐transcribed into cDNA using the SuperScript III Reverse Transcriptase Kit (#18080044; Invitrogen) following the standard protocol. The gene‐specific primers used were the 515F (sequence: GTGCCAGCMGCCGCGGTA) and 806R primer (sequence: GGACTACHVGGGTWTCTAAT) for amplification of the V4 region of the bacterial and archaeal 16S rRNA gene, as provided by the Earth Microbiome Project (Apprill *et al*., 2015). The cDNA served as the template for PCR amplification: each 25 μl reaction volume contained 12.5 μl of HiFi Master Mix (M049S; New England BioLabs; Ipswich, MA, USA), 10 μl of PCR‐grade H_2_O, 0.5 μl of 10 μM 806R reverse primer, 1 μl of 5 μM barcoded 515F forward primer, and 1 μl of cDNA template. The following thermocycler conditions were used: an initial denaturation at 98°C for 3 min, followed by 35 cycles of denaturation at 98°C for 30 s; annealing at 55°C for 30 s and extension at 72°C for 30 s, with a final extension at 72°C for 7 min; and a final hold at 10°C. Technical replicates were combined before amplicon concentrations were determined using the PicoGreen dsDNA Assay Kit (#254406, Invitrogen), performed on the Tecan Infinite® 200 PRO M Plex microplate reader (Männedorf, Switzerland). Samples were normalized, pooled, and sequenced on a MiSeq i100 Benchtop Sequencer by Illumina (San Diego, CA, USA) using the custom primer protocol with the NextSeq 1000/2000 XLEAP‐SBS Read & Index Primer Kit (#20112856; Illumina), PhiX Control v3 (FC‐110‐3001; Illumina) spiked in at 30%, and the MiSeq i100 Series 25M Reagent Kit (300 cycles, #20126568; Illumina). Fastq files were generated on the Illumina secondary analysis platform BaseSpace using the DRAGEN BCL Convert 4.3.13 application. Genus‐level total read counts from the active 16S amplicon sequencing are available in Dataset S2.

### RNA library prep/sequencing

Extracted RNA was sent to Novogene (Sacramento, CA, USA) for library preparation and sequencing. Ribosomal RNA (rRNA) was removed using a proprietary rRNA depletion kit (Novogene). Libraries were constructed with the ABclonal® Fast RNA‐seq Library Prep Kit V2 for Illumina (ABclonal, Woburn, MA, USA) according to the manufacturer's protocol, using both nondirectional (default) and directional workflows. Libraries were sequenced on the Illumina NovaSeq X Plus platform (Illumina).

### Bioinformatic workflow – amplicon

For both 16S amplicon sequences from DNA and RNA, demultiplexed sequence data were processed in RStudio, following the Dada2 v.1.34.0 pipeline to infer amplicon sequence variants. Based on the quality score, the 16S reads from the DNA forward and reverse strands were truncated to 300 and 285 nucleotides, respectively. The 16S from RNA forward and reverse reads were both truncated at position 150. Forward and reverse reads were merged, chimeras were removed, and taxonomy levels were assigned using the SILVA NR99 16S rRNA To Species Training Database v.138.2 (Quast *et al*., 2013; Glöckner *et al*., 2017).

Reads mapping to mitochondria or chloroplasts were filtered, and taxa present in fewer than two samples or with fewer than 10 reads in at least two samples were omitted to remove low‐abundance and rare taxa. Alpha diversity metrics were computed using the phyloseq package (McMurdie & Holmes, 2013). As above, read counts were converted to relative abundance, and the center log‐ratio (CLR) was computed. The CLR‐transformed data were used to generate a Euclidean distance matrix for beta‐diversity estimation using the phyloseq R package. Differences between samples in the distance matrix explained by temperature, microbiome, and genotype were tested for statistical significance using a permutational multivariate analysis of variance (PERMANOVA) in the vegan v.2.6‐10 R package, with 999 permutations (Jari *et al*., 2025).

To compare DNA vs RNA amplicon sequencing, samples were rarified to 50 K reads using the phyloseq package. A mean value for each taxon was calculated at each level of Genotype, Temperature, and microbiome complexity. Spearman correlations were calculated for each comparison of taxa abundances and standard deviation. A one‐sided *t*‐test was used to determine whether the slope was significantly less than the expected value (1).

### Bioinformatic workflow – metatranscriptome

Adapters and low‐quality reads were trimmed from the raw Fastq files using Trimmomatic v.0.39 (Bolger *et al*., 2014). The Hisat2 v.2.2.1 aligner was used to filter reads that aligned with the human genome (Kim *et al*., 2019; Morales *et al*., 2022). Any sequenced rRNA was identified and removed using a deep learning method implemented in Ribodetector v.0.3.1 (Deng *et al*., 2022). After preprocessing, filtered sequences were aligned to the B73 v.5 (maize) or BTx623 v.3 (Sorghum) using Hisat2 (Hufford *et al*., 2021; Wang *et al*., 2021). Aligned reads were assigned a feature by the featurecounts tool from Subread v.2.0.6 (Liao *et al*., 2014). For the cross species comparison, orthologous genes between the references B73 and BTx623 were identified using orthofinder v.2.5.5 (Emms & Kelly, 2019). Gene counts were assigned to orthogroup groups (including 1 : 1 and 1 : many relationships) using a custom R script. To normalize the model across species and account for differences in genomic structure and basal transcription rates, transcript abundance was scaled using a median‐of‐ratios before ortholog assignment. Lowly expressed genes were removed (< 75 reads in < 4 samples) before differential expression analysis of maize or sorghum genes conducted using DESeq2 v.1.48.1 in R v.4.5.0 using the model Expression = Genotype + Temperature + Microbiome + Microbiome : Temperature (Love *et al*., 2014; R Core Team, 2021). Gene Ontology (GO) enrichment analysis was performed using the Ensembl Plants database and the biomart v.2.64.0 and topgo v.2.60.1 R packages (Kinsella *et al*., 2011; Yates *et al*., 2022; Alexa & Rahnenfuhrer, 2025).

Microbiome composition was inferred from reads that did not align to the maize genome by comparing sequences to the RefSeq database using kraken2 v.2.14 (O'Leary *et al*., 2016; Wood *et al*., 2019). A KEGG function was assigned to each read using the diamond v.2.1.8 tool and the eggnog v.1.12 mapper, and the eggnog v.5.0 database, including fungal, bacterial, and archaeal orthologs (Huerta‐Cepas *et al*., 2019; Buchfink *et al*., 2021; Kanehisa *et al*., 2025). Microbial genes annotated via EggNog were filtered to remove low‐confidence genes (e‐value < 1e‐6), then collapsed into pathways by EC number and assigned functional annotations from the KEGG database using the keggrest v.1.42.0 and tidyverse v.2.0.0 packages in R (Wickham *et al*., 2019; Tenenbaum & Maintainer, 2024).

Low‐abundance microbial genes were filtered before analysis, retaining only those with at least 250 reads in three or more samples. Because the number of microbial transcripts captured in each library likely represents only a subset of the total root transcriptome, expression values were normalized to relative abundance to account for differences in sequencing depth and microbial representation among samples. Read counts were subsequently transformed using the centered log‐ratio (CLR) method implemented in the compositions R package (v.2.0‐8) to address the compositional nature of the data (van den Boogaart *et al*., 2025). PCA was performed on CLR‐transformed data to visualize overall transcriptional profiles across samples. Samples were grouped by genotype and microbiome complexity (intact or depleted), and differential expression analysis was conducted using Aldex2 (v.1.40.0) to identify microbial genes whose expression differed in response to temperature (Fernandes *et al*., 2013). Aldex2 was selected for its ability to handle the compositional structure and sampling variability inherent to microbial metatranscriptomics data (Gloor *et al*., 2016). For both gene‐level and pathway‐level analyses, raw count matrices were first subjected to Monte Carlo (MC) sampling from a Dirichlet distribution (*n* = 228 instances per sample) to model technical variation in the dataset. A CLR, transformation was applied to each instance to prepare the compositional data for linear modeling. To evaluate experimental factors on pathway abundance, we employed a general linear model (GLM) with the following design: (Expression = Genotype + Temperature + Microbiome + Microbiome : Temperature). Effect sizes were defined as the expected values of the regression coefficients. Significance was determined using Benjamini‐Hochberg (BH) adjusted *P*‐values (*q* < 0.05). For gene‐level differential expression (DE), pairwise comparisons between temperature treatments within specific microbiome/genotype groups were performed using the aldex.glm.effect and aldex.glm functions to estimate the magnitude of change and associated false discovery rates (FDRs).

### Bioinformatic workflow – coexpression

To assess coexpression, we analyzed various features of the plant and microbiome transcriptome. The following analyses were conducted in parallel using gene‐level microbiome transcription data and collapsed KEGG pathways. A Mantel test was used to examine the relatedness of plant and microbiome expression profiles by computing distance matrices of plant (median‐to‐ratio‐transformed) and microbial (CLR‐transformed) transcript abundance datasets with the vegan v.2.6‐10 package's Mantel command, using a Spearman correlation and 5000 permutations. PERMANOVA tests were conducted for both the plant and microbial distance matrices to evaluate the contributions of genotype, microbiome complexity, and temperature to variation in gene expression.

We conducted a differential correlation analysis to compare pairwise co‐expression patterns at 28°C and 38°C. Pairwise Spearman correlations were then computed between all plant genes and microbial features within each temperature condition, excluding intradomain correlations (i.e. only plant–microbe pairs were tested). For each pair of plant and microbial genes, Fisher's *z*‐transformation was applied to correlation coefficients at both temperatures, and the difference between *z*‐scores was used to quantify changes in association strength between 28°C and 38°C. The standard error of the difference was calculated assuming independent sampling, and a two‐tailed *z*‐test was used to determine statistical significance. FDR correction was applied using the Benjamini–Hochberg method, and significant differential correlations were defined as those with FDR < 0.05 and |Δ*z*| > 1.

To identify plant genes and microbial pathways most strongly associated with key traits, we applied two complementary predictive modeling approaches: elastic net regression and random forest. Before modeling, plant and microbial abundance matrices were scaled, and features with weak univariate correlations (Spearman's ρ >0.05) with the response variable were filtered out to reduce dimensionality. For elastic net regression, models were fit separately for biomass and heat stress using a 50 : 50 mixture of L1 and L2 penalties (α = 0.5) using the glmnet v.4.1‐10 R package (Tay *et al*., 2023). Ten‐fold cross‐validation was performed to select the optimal regularization parameter (λ), and nonzero coefficients were extracted to identify features contributing most strongly to trait variation. Features were annotated by type (plant gene or microbial pathway), and separate columns were retained for biomass and heat stress‐associated coefficients.

Random forest models were fit to the same filtered datasets using 2000 trees and 10‐fold cross‐validation in the randomforest v.4.7‐1.2 R package (Breiman *et al*., 2024). Variable importance was calculated for each feature, and the top 25 plant genes and microbial pathways were extracted for each trait.

Results were summarized to identify features consistently represented across modeling approaches, enabling the integration of co‐expression analysis methods to highlight plant–microbe interactions linked to heat response phenotypes.

### Bacterial taxonomic tree generation

To visualize the phylogenetic distribution of bacteria, a taxonomic tree was constructed from a list of all microbes identified by the EggNog collapsed at the family level using the taxize R package (Chamberlain & Szöcs, 2013). Unique taxa were queried against the NCBI database to retrieve their full hierarchical lineage, and the tree was generated from these lineages using the *class2tree* function. The resulting phylogeny was visualized in the Interactive *Tree of Life* (iTol) v.6 (Letunic & Bork, 2007). Families were annotated by taxonomic class, with additional rings representing global family‐level transcription shifts between 28°C and 38°C (Wilcoxon rank‐sum test, *P* < 0.05) and the counts of DAA metabolism genes in each family differentially correlated between temperatures (Fisher's *Z*‐transformation of Spearman rho coefficients, FDR < 0.05).

All data visualization was conducted in R using the ggplot2 v.3.5.1 and ggpubr v.0.6.0 packages (Wickham, 2011; Kassambara, 2023).

## Results and Discussion

### Plant phenotype analysis

**Fig. 1:**
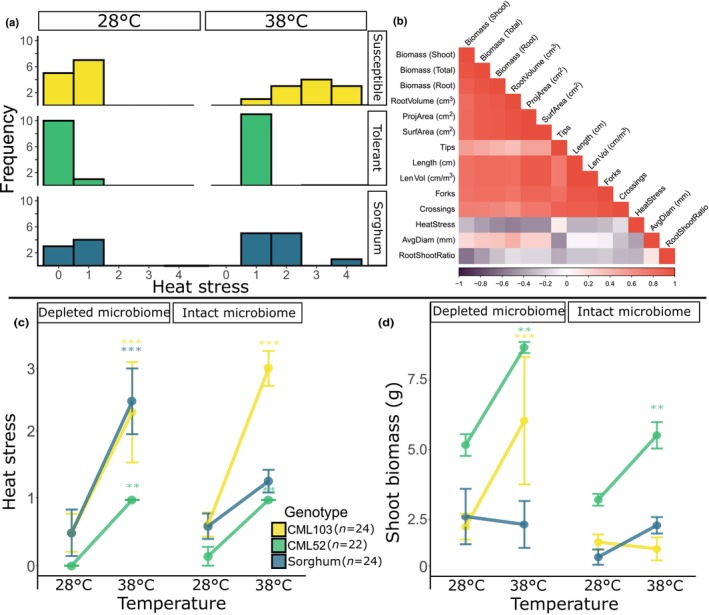
Plant phenotype analysis. The categorical heat stress score was distributed as expected across genotypes (a). The heat stress score was negatively correlated with plant biomass and root phenotypes, which were highly correlated with each other (b; Spearman's rho). Impact of microbiome complexity (depleted vs intact) and temperature on heat stress scores (c) and shoot biomass (d). Points (in c, d) represent mean values and error bars represent the SE. Total sample size for each genotype is indicated in the legend. Asterisks denote significant differences in temperatures within each microbiome and genotype combination determined by Tukey's HSD (**, *P* < 0.01; ***, *P* < 0.001).

The plant genotypes responded as expected to heat stress, with survival rates > 90% across genotypes. Plants that died before the end of the experiment were assigned a heat stress value of 4 and excluded from RNA sequencing. The heat‐tolerant maize genotype, CML52, exhibited the lowest heat stress scores at 38°C, whereas the heat‐susceptible maize genotype, CML103, showed the highest, and the sorghum genotype displayed an intermediate phenotype (Fig. 1a). To verify the heat stress score, we treated it as a numeric variable and found it to be negatively correlated with biomass and most root phenotypes (Fig. 1b). We observed significant effects of both microbiome complexity and temperature on the heat stress score (Fig. 1c). While CML52's heat response remained consistent across microbiome complexities, its shoot biomass was significantly higher with a depleted microbiome (Wilcoxon, *P* = 0.017).

With an intact rhizosphere microbiome, CML103's heat stress score at 38°C was higher than when the microbiome was depleted. Interestingly, the sorghum genotype exhibited the opposite trend, with a higher heat stress score with a depleted microbiome at 38°C compared to an intact microbiome. The effect of microbiome complexity on shoot biomass and root volume also showed contrasting trends between CML103 and the sorghum genotype, where sorghum appears to be more protected from heat stress with an intact microbiome, and CML103 is more protected in a depleted microbiome (Figs 1d, S2A). Unlike the qualitative heat stress score and aboveground or belowground biomass, the root‐to‐shoot ratio, calculated from biomass, consistently decreased in response to the high‐temperature treatment similarly across genotypes in both levels of microbiome complexity, consistent with the literature (Fig. S2B) (Calleja‐Cabrera *et al*., 2020). The negative correlation between biomass and root‐to‐shoot ratio is driven by this temperature‐induced shift in biomass partitioning where plants maintain a higher ratio of root biomass under optimal conditions. In the more heat‐tolerant genotypes, there is also an increase in total biomass, leading to an inverse relationship. We used these four phenotypes (quantitative heat stress score, shoot biomass, root volume, and root‐to‐shoot ratio) as biomarkers of heat stress.

There is a high degree of variation in CML103 phenotypes at high temperatures with a depleted microbiome (Fig. 1c,d). However, this observation is not unexpected; many microbiome treatments in otherwise identical samples result in varied phenotypes, referred to as the responder and nonresponder phenomena in animal systems (Diener *et al*., 2021). Though attention is not typically drawn to unexplained variation, its presence in plant studies underscores that the effects of microbiome perturbation are not always uniform (Li *et al*., 2025; Olimi *et al*., 2025). To address the issue of responder/nonresponder, most downstream analyses were conducted to identify plant genes and microbial composition or function that impact the plant phenotype, agnostically of the experimental design.

The results demonstrate that the magnitude and direction of a plant's response to heat stress depend not only on the plant's genotype, abiotic environment, and the G × E interaction but also on the underlying context of the rhizosphere microbiome. These findings underscore the power of the GERMs framework in elucidating complex plant phenotypes. Identifying these complex interactions is the first step in uncovering the microbiome‐mediated mechanisms by which plants adapt to stress.

### System‐level metatranscriptomic sequence assignment

Reads from each sample were parsed into those belonging to maize/sorghum, bacteria, or fungi after removal of human reads and rRNA. Of an average read count of 57.7 M (range 41.7–81.1 M), *c*. 3.4 M (range 0.62–9.5 M) reads were aligned to the maize B73 genome in maize samples, whereas *c*. 3.2 M (range 1.4–7.1 M) reads were aligned to the sorghum Tx623 genome in sorghum samples. The remaining reads were assumed to be microbial and were assigned a function and binned into those belonging to bacteria (average 22.7 M, range 9.4–53.14 M) or fungi (average 2.1 M, range 0.31–5.7 M) using the EggNog database (e‐value < 1e−6). Reads were given a lower taxonomic assignment based on sequence similarity to genomes in the RefSeq database (Fig. S3). The complete list of per‐sample read counts is available in Table S3.

Mapped read counts to maize and sorghum features varied widely and were below optimal coverage. Improved root cleaning before sonication could increase the microbial‐to‐plant read ratio, and higher sequencing depth per sample would further mitigate this limitation. Less stringent alignment might recover more plant features but risks misassigning fungal reads to plants. Low plant read counts can bias analyses of low‐abundance features; accordingly, we applied a conservative filter to remove genes with low expression (< 75 reads in < 4 samples). For bacteria and fungi, we applied a stringent *e*‐value cutoff (< 1e−6) in EggNog to reduce false positives and misassigned plant reads (Fig. S3). These criteria yielded a substantial fraction of unknown reads, some attributable to protists and viruses, which were retained as unknown because they were outside the study's scope. Unknown metatranscriptomic reads in this study, and others, may harbor valuable information that could be revisited as annotation tools and databases improve.

### Rhizosphere microbial composition and comparison of sequencing approaches

Microbial community composition was calculated from three sources: active amplicon (16S from RNA), complete amplicon (16S from DNA), and transcriptomic sequencing. Beta‐diversity analysis, conducted independently for each sequencing type, revealed highly similar patterns of microbiome composition driven by microbiome complexity and temperature treatment (Fig. S4). Each dataset was trimmed and collapsed at the genus level. Read counts for the transcriptomic dataset were up to 100‐fold higher (*c*. 15 M) than for either the active amplicon (250 K) or the complete amplicon (156 K). For a fair comparison between sequencing types, samples were rarified to a minimum read count of 50 K. After filtering and rarefaction, 356 genera were assigned in the active amplicon dataset, 336 in the complete amplicon dataset, and 913 bacterial genera in the transcriptome dataset (Fig. S5A). A shared set of 164 genera represented the majority of the community's relative abundance in the complete amplicon (78%), the active amplicon (68%), and the transcriptomic data (65%).

Transcriptomic data also seemed to capture more variation due to host genotype than either amplicon dataset (Fig. S4D). When comparing shared genera at the mean value for each genotype‐treatment level, we observed linear relationships between sequencing types. However, comparisons involving the transcriptomic dataset were right‐skewed, indicating that low‐abundance taxa are better captured by transcriptomic sequencing (Fig. S5B). Similarly, we observed lower variation between biological replicates in the transcriptomic dataset than in either amplicon dataset (Fig. S5C). The two amplicon datasets were most similar to each other, with slightly greater variability in the active 16S dataset than in the complete 16S dataset.

The lower variation in taxonomic assignment in the transcriptomic dataset may be partly due to its higher resolution compared to the amplicon dataset. Many genera identified in the transcriptomic dataset could not be distinguished by amplicon sequencing. For example, amplicon sequencing grouped *Paraburkholderia*, *Burkholderia*, and *Caballeronia*, which the SILVA database annotates as *Burkholderia–Caballeronia–Paraburkholderia*, whereas the transcriptomic dataset identified each genus separately. These genera are all members of the *Burkholderia sensu lato* group that share 96–100% sequence similarity of their published full‐length 16S rRNA genes. Yet key functional differences exist among *Burkholderia sensu lato* members, including their roles in nitrogen and carbon cycling (Bach *et al*., 2023).

Although we expected greater similarity between the active 16S and transcriptomic datasets, the two amplicon datasets were most similar to each other. This observation is likely driven by amplification and database bias, as both amplicon datasets were assigned taxonomy using the SILVA database, whereas the transcriptomics data were assigned using RefSeq. In almost all metrics (number of genera identified, taxonomic relative abundance, and replicate variance), the amplicon datasets performed similarly. However, among the 164 taxa that overlapped across the datasets, they accounted for a greater proportion of total abundance in the complete amplicon dataset than in the active amplicon and transcriptomics datasets (78% vs 68% and 65%). This indicates that while the set of taxa identified by all three sequencing types dominates the community, activity, and gene expression are more broadly distributed across both abundant and rare microbes, suggesting functional contributions are not proportional to microbial relative abundance.

The metatranscriptomic dataset provides both higher taxonomic resolution and functional insight, capturing the same community structure observed by amplicon sequencing while extending it to reveal which members are transcriptionally active and which pathways are enriched. Although more time‐ and resource‐intensive, metatranscriptomics offers a comprehensive view of microbiome composition and function, making it an extremely informative approach for dissecting plant–microbe interactions.

### Maize and sorghum differential expression analysis

**Fig. 2:**
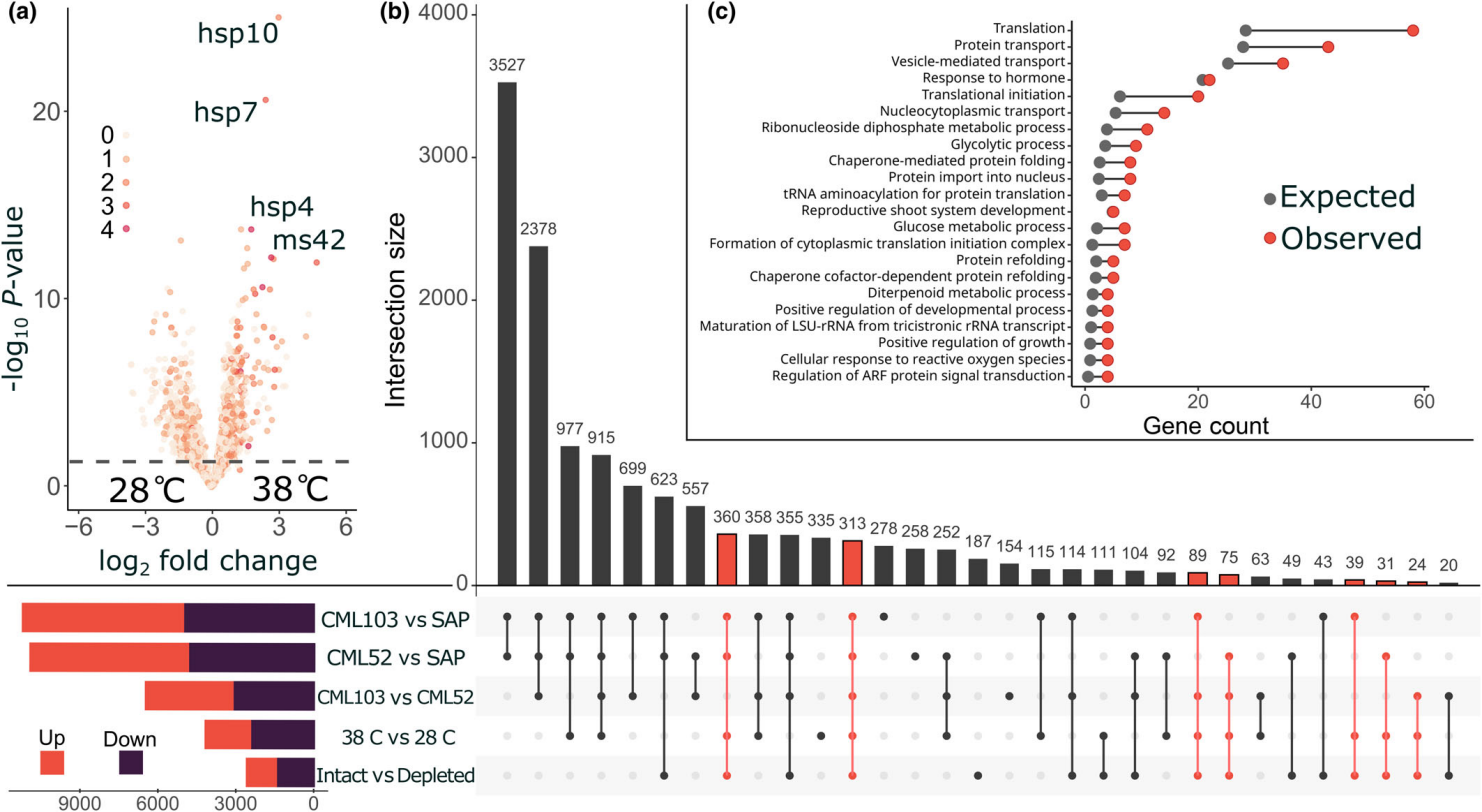
Shared and divergent heat stress responses in *Zea mays* (CML52 and CML103) and *Sorghum bicolor* (SAP). Differentially expressed genes under heat stress from a combined maize–sorghum ortholog dataset are displayed in a volcano plot, with points colored by the number of publicly available datasets in which they appear. Key heat‐response genes, including multiple heat shock proteins (hsp4, hsp7, and hsp10) and a mitochondrial stress protein (ms42), are strongly upregulated and consistently recovered across datasets (a). A large number of differentially expressed genes were identified between the genotypes. To narrow our focus, we selected 931 genes that were differentially expressed (highlighted in red) when comparing temperatures, microbiome complexity, and genotype independently (b). GO term enrichment in the list of intersecting genes differentially expressed due to temperature, microbiome complexity, and genotype (c).

We compiled a joint maize–sorghum transcriptome dataset restricted to orthologous genes, with samples averaging 2.7 million reads assigned to orthologous genes. A complete list of orthologs used in this study is included in Table S4. To confirm that combining datasets did not obscure the heat stress response, we compared our results with those from published maize and sorghum heat stress transcriptomic studies. Across six independent studies spanning multiple tissues and developmental stages, 67 genes were consistently differentially expressed in response to heat stress. Of these, 36 genes (53.7%) were also identified in our dataset (Fig. 2a), including genes with demonstrated roles in heat tolerance: *hsp4*, *hsp7*, *hsp10*, and *ms42* (Li *et al*., 2022; Diogo *et al*., 2023). Details of these studies, along with a complete list of identified genes, are available in Datasets S3 and S4 (Johnson *et al*., 2014; Li *et al*., 2020; Yang *et al*., 2022; Ali *et al*., 2025; Wang *et al*., 2025).

Differential expression analysis revealed extensive transcriptional divergence between genotypes, particularly between species (Fig. 2b). To narrow the focus to genes involved in microbiome regulation in response to heat stress, we selected intersecting genes, those differentially expressed across genotype, temperature, and microbiome complexity comparisons. Of the total list of *c*. 13 000 genes differentially expressed in at least one comparison, 931 intersected all three (Fig. 2b highlighted in red).

Functional enrichment analysis of these intersecting genes revealed a significant overrepresentation of translation‐ and transport‐related processes, as well as chaperone‐mediated protein folding and hormone responses (Fig. 2c; Dataset S3). These functions indicate a role of protein homeostasis and stress signaling in the microbe‐mediated, shared maize–sorghum heat response.

Our analysis used orthologous genes between maize and sorghum to efficiently integrate datasets. Noting that while syntenic orthologs are more likely to have conserved functions, including all orthologs allows us to capture genes with species‐specific functional and expression differences that may be relevant under different microbiome complexities and high temperatures (Zhang *et al*., 2017; Adhikari *et al*., 2021).

### Microbial gene and pathway enrichment

Microbial genes annotated by EggNOG showed global microbial gene expression was driven by microbiome complexity and temperature (Fig. 4a (see later), PERMANOVA: Microbiome = 0.38, *P* < 0.001, Temperature = 0.13, *P* < 0.001). Though these environmental parameters dominated the overall community gene expression, host genotype was also a significant modulator of the microbial temperature response. 6258 of 27 911 microbial genes were significantly differentially expressed in response to temperature across at least one genotype‐by‐microbiome grouping (Fig. S6A). The expression patterns show some similarities across groups but reveal unique expression profiles driven by both microbiome complexity and genotype.

Microbial pathways were consistently differentially expressed in response to microbiome complexity and, in many cases, to temperature. Quantification of the main and interaction effects using an ALDEx2 GLM revealed that microbiome complexity was the dominant factor influencing the relative abundance of many metabolic pathways (Fig. S6B). Specifically, the Microbiomecontrast exhibited the largest effect sizes (standardized coefficients; see the Materials and Methods section) across several top pathways, including the biosynthesis of macrolides and polyketides, underscoring the overriding importance of the soil environment in shaping microbial functional potential. Pathways such as furfural degradation and flavonoid biosynthesis showed strong effect sizes driven by the Microbiomex Temperature interaction, suggesting a condition‐specific mechanism for the microbial degradation of aromatic and toxic compounds. The significant effect sizes observed in the biosynthesis of several antibiotic classes, including macrolides, tetracycline, and novobiocin, indicate that microbiome complexity is necessary for transcription of microbial secondary metabolite gene clusters. These shifts in antimicrobial biosynthesis likely reflect a dynamic, environmentally driven competition in the assembly and functional stability of the root‐associated microbiome.

### Plant gene and microbial pathway coexpression

**Fig. 3:**
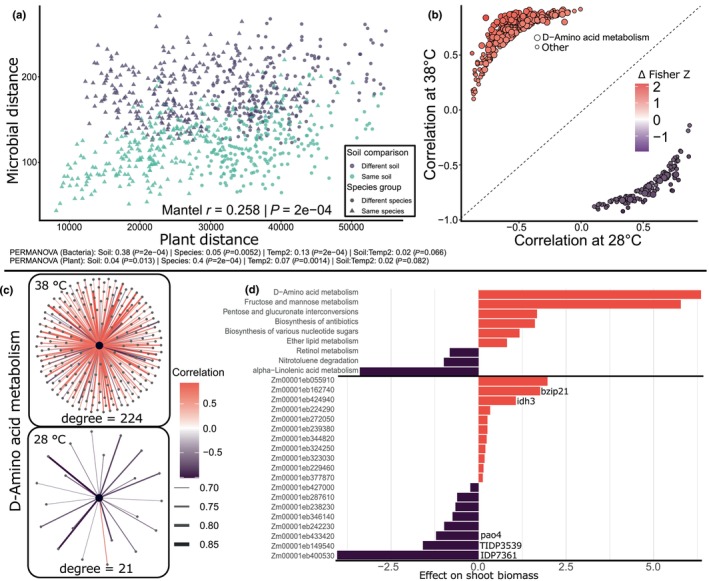
Co‐expression between plant and microbial transcriptional profiles. Mantel test comparing pairwise distance matrices of plant and microbial gene expression, revealing significant co‐variation between host and microbial transcriptomes. Clustering and PERMANOVA analyses indicate data are structured by microbiome complexity and host species (a). Differential bipartite correlation analysis showing the correlation values (Spearman's ρ) for microbial pathway–plant gene pairs at high and optimal temperatures. Only points that were significantly differentially correlated (FDR < 0.05) are shown on the plot, colored by change in *Z* score (b). Bipartite network showing d‐amino acid metabolism pathway and connections (Spearman's ρ >0.7) to plant genes at optimal conditions and under heat stress (c). Elastic net regression identifies microbial pathways and plant genes most strongly associated with variation in biomass. Bars represent feature coefficients; positive values indicate increased expression associated with greater shoot biomass (d).

To quantify the linear association between the host and microbiome functional profiles, we performed a Mantel test on distance matrices of plant and microbial gene expression. This analysis revealed significant covariation between host and microbe transcriptomes (Mantel *r* = 0.258, *P* = 2e‐04; Fig. 3a), demonstrating that changes in the microbiome transcriptome mirror shifts in plant gene expression. However, clustering of points in the Mantel test revealed relationships in the data driven in part by microbiome complexity and host species. To estimate the contribution of host species, temperature, and microbiome complexity to gene expression, we conducted a PERMANOVA analysis to assign numerical values to each variable for both plant and microbial gene expression. In both PERMANOA analyses, all main effects were statistically significant (*P* < 0.05). Microbiome complexity had a much greater effect on microbial gene expression, and host species had a greater effect on plant gene expression (Fig. 3a).

To further explore specific coregulated functions that responded to high temperatures, we constructed a bipartite co‐expression network linking plant genes and microbial pathways at both optimal and high temperatures and conducted a differential correlation analysis to identify relationships between plant genes and microbial pathways that are different between temperatures (Δ Fisher *Z* ≥ 1 and FDR < 0.05; Fig. 3b). The results of this analysis, shown in Fig. 3b, demonstrate that most differentially correlated pairs fall in the upper‐left quadrant, indicating that more plant–microbe relationships shifted from negative or weak associations under optimal conditions to strong positive correlations under heat stress (361 pairs) than shifted toward negative correlations (131 pairs). Under heat stress, clusters of genes and pathways that are often lowly expressed under optimal conditions are upregulated. Of 492 correlated pathway and gene pairs, 245 contained the microbial DAA metabolism pathway. Microbial DAA metabolism exhibits a moderate degree of mostly negative correlations with plant genes under optimal conditions, whereas under heat stress, it shows a high degree of positive connectivity with maize gene expression (Fig. 3c).

We employed elastic net regression to associate transcriptional features with phenotypic outcomes, identifying both plant genes and microbial pathways predictive of phenotypic variation in biomass, root volume, and heat stress tolerance (Fig. 3d). The elastic net model identified 11 microbial pathways and 31 plant genes as explanatory of plant biomass; 8 microbial pathways and 39 plant genes for root volume; 2 microbial pathways and 49 plant genes for root shoot ratio; and 5 microbial pathways and 28 plant genes for heat stress.

Of the plant genes associated with heat‐tolerance biomarkers a putative palmitoyl protein thioesterase (*Zm00001eb055910*) is one of the strongest positive predictors of biomass. As a regulator of protein depalmitoylation and membrane‐associated signaling complexes, it plausibly influences root surface communication with the microbiome during heat stress. Two additional genes, *bZIP21* and *IDH3*, were also positively correlated with biomass. *bZIP21* integrates reactive oxygen species (ROS), ABA, and carbon allocation signals and may help maintain coordinated host–microbe signaling under stress. While *IDH3*, a mitochondrial NAD‐dependent isocitrate dehydrogenase subunit, supports TCA cycle activity and organic acid production, functions consistent with enhanced metabolic capacity in heat‐tolerant plants.

Conversely, *PAO4*, *TIDP3539*, and *IDP7361* were negatively associated with biomass. *PAO4* mediates polyamine catabolism, a pathway broadly activated under cellular stress; *TIDP3539* encodes an RNA‐binding protein likely involved in posttranscriptional stress regulation; and *IDP7361* (mannose‐1‐phosphate guanyltransferase) contributes to GDP‐mannose biosynthesis linked to cell‐wall modification. Reduced expression of these stress‐associated genes in high‐biomass plants indicates a shift away from generalized stress signaling and toward metabolic efficiency and microbially supported growth.

Similar to the elastic net analysis, a random forest approach was applied to identify features that explain plant biomass, root volume, and heat stress. When both microbial pathways and plant genes were included, plant genes were rated higher variable importance than microbial pathways in all tests (Fig. S7). To identify a shortlist of candidate genes and pathways, the results of the three analyses were compiled, and features identified in two or more models were selected (Fig. S8; Table S5).

The same three analyses were conducted using gene‐level microbial data, with similar results, and generated a list of 73 candidate microbial genes and 81 plant genes identified by at least two tests (Table S5). Notably, two microbial genes, *amt* from *Sphingobacteriales* and *gcvT* from *Planctomycetota*, were identified in four of the nine tests. Members of the order *Sphingobacteriales* are frequently implicated in plant stress responses (Hagaggi & Abdul‐Raouf, 2022). Differential regulation of the *Sphingobacteriales amt* ammonium transporter suggests that nitrogen cycling may underlie *Sphingobacteriales*' involvement in host stress biology. Similarly, the *gcvT* gene encodes a glycine cleavage T protein involved in C_1_ metabolism and amino‐acid turnover.

The candidate maize, sorghum ortholog supported by the most analyses is *Zm00001eb373970*, named *arftf27* or *aux16*, an annotated auxin‐signaling transcription factor. This gene has previously been associated with environmental stress and root morphology, but not with microbiome modulation, even though auxin signaling is a primary mode of communication between plants and their root microbiomes (Saidi & Hajibarat, 2020; Zhang *et al*., 2024).

Several microbial pathways and maize gene relationships identified through co‐expression analysis appear biologically plausible, particularly those linking microbial carbon and sugar metabolism (e.g. pentose and glucuronate interconversions, fructose and mannose metabolism, and starch and sucrose metabolism) with plant genes involved in the biosynthesis and transport of sugars and amino acids (Fig. S8). Such coupling is consistent with increased rhizodeposition under heat stress, in which elevated root exudation could promote the expression of carbon‐catabolizing enzymes by microbes. Similarly, microbial retinol metabolism and terpenoid backbone biosynthesis were correlated with maize transcription factors associated with oxidative stress (e.g. ethylene‐responsive and peroxidase genes), suggesting a shared role in modulating reactive oxygen balance or lipid‐derived signaling during heat adaptation.

The presence of microbial DAA metabolism, coupled with plant genes related to protein modification and turnover (e.g. serine/threonine kinases, SUMO‐conjugating enzymes, and REF6 demethylase), may reflect active exchange or sensing of DAA in the rhizosphere. We focused on this pathway because, although DAA have been reported to exert both positive and negative effects on plants, the highly correlated expression profiles suggest a novel finding: that these molecules may participate directly in plant–microbe synergistic responses to abiotic stress (Hill *et al*., 2011; Gu *et al*., 2020; Porras‐Dominguez *et al*., 2024).

By integrating plant and microbial metatranscriptomes into a single framework, we can identify co‐expression patterns of microbial and plant functions that were not apparent when each dataset was analyzed independently. Treating the plant and rhizosphere transcriptomes as interacting systems that contribute equally to the host phenotype has uncovered coordinated host–microbe responses to heat stress and provides a unique window into the functional coupling between plants and their rhizosphere microbiome under abiotic stress.

### Evidence for a plant‐mediated DAA metabolism shift under heat stress

**Fig. 4:**
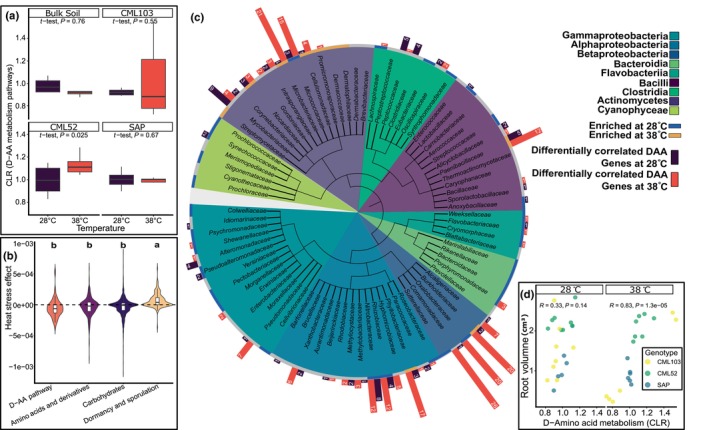
D‐Amino acid (DAA) metabolism dynamics across soils, temperatures, and maize genotypes. Centered log‐ratio (CLR) abundance of DAA metabolism pathways in bulk soil and the rhizospheres of three genotypes at 28°C and 38°C. DAA metabolism remained unchanged in bulk soil exposed to heat stress, but significantly increased in the rhizosphere of heat‐tolerant CML52 (a). The effect of heat stress on microbial metabolic pathways in bulk prairie soil across > 30 locations, derived from publicly available data. The DAA pathway exhibited a negative shift under heat stress, in contrast to the positive increases observed in the dormancy and sporulation pathways (letters denote significant differences by Fisher's least significant difference, *P* < 0.05; the dashed line is drawn at 0, the value given if there were no differences between gene abundance due to heat stress) (b). For boxplots in (a) and (b) the internal horizontal line represents the median, boxes are draw to the 25^th^ and 75^th^ percentile and whiskers represent 1.5 times the interquartile range. Phylogeny of bacterial families identified in the transcriptome analysis, colored by taxonomic class. The innermost ring indicates families enriched at 28°C (blue) or 38°C (orange). The outermost ring shows the number of DAA metabolism genes differentially correlated with maize genes at 28°C (purple bars) and 38°C (red bars). Spearman correlation between DAA metabolism (CLR) and root volume (cm^3^) under standard (28°C) and heat stress (38°C) conditions (c). Under high temperatures, DAA metabolism is highly correlated with root volume (*R* = 0.83, *P* = 1.3e‐05) (d).

The observed correlation between microbial DAA metabolism and plant performance was genotype, temperature, and microbiome‐dependent (Fig. 4a,d). The most consistent increase was in the heat‐tolerant CML52, and the strongest increase was observed in CML103 individuals that displayed high biomass and a depleted microbiome (Figs 1a, 4d). This pattern suggests that the upregulation of microbial DAA metabolism is associated with plant performance under heat stress. While the bulk soil control (no plant) showed no corresponding increase in DAA metabolism and trended slightly downward, a meta‐analysis of a publicly available metagenomic dataset from 30 grassland soils revealed a decrease in DAA metabolism under experimental heat stress compared to the microbial stress response (dormancy and sporulation) in line with total amino acid and carbohydrate metabolism (Fig. 4b) (Knight *et al*., 2024). This observation is consistent with previous findings that DAA cycling tends to decline when microbial communities are directly stressed (Porras‐Dominguez *et al*., 2024).

Maize and sorghum genes significantly correlated with microbial DAA metabolism support a signaling‐based hypothesis (Table S5). Only 58 of the 224 plant genes correlated with DAA metabolism were also differentially expressed under heat stress. This is significantly fewer than expected if both responses were independently driven by temperature (one‐tailed hypergeometric test, *P* < 0.01), suggesting that DAA metabolism and plant gene expression are functionally coupled rather than parallel responses to temperature. Highly correlated plant genes included key carbon metabolism enzymes, such as invertase2 (*ivr2*), aldolase1 (*ald1*), enolase1 (*eno1*), and phosphoenolpyruvate carboxylase4 (*pep4*), consistent with increased metabolic flux and carbohydrate mobilization, which could alter carbon exudation to stimulate rhizosphere colonization. Upregulation of redox‐regulatory and calcium‐signaling genes (*nucleoredoxin1*, *glutathione reductase2*, *prdx4*, *cipk20/33*, *annexin1/2*) suggests a possible ROS‐activated signaling and transport network. The co‐expression of transcription factors possibly linked to stress signaling (*WRKY*, *CAMTA*, and *ARF* families) could indicate integration of DAA‐associated cues into regulatory pathways. Structural remodeling at the root–microbe interface may also be involved, given correlations with cellulose synthase8 (*cesa8*), xyloglucan endotransglucosylase/hydrolase7 (*xth7*), and root hair defective3 (*rth3*). Multiple of these gene types in maize and sorghum have previously been reported to affect microbiome function, particularly root hair formation by *rth3* and sugar allocation by *ivr2* (Sun *et al*., 2020; Korth *et al*., 2024; Hartwig *et al*., 2025).

### DAA gene‐level analysis and taxonomic distribution

To identify the specific microbial drivers, we re‐analyzed genes annotated to DAA metabolism using the same tri‐analysis (differential correlation, elastic net, and random forest). This approach identified two high‐confidence candidate genes (identified in four of nine tests): *S‐adenosylmethionine decarboxylase* (*speH*) from *Burkholderiaceae* and *Branched‐chain‐amino‐acid aminotransferase* (*ilvE*) from *Streptosporangiales*. Genes identified in at least two of nine analyses mirror functional patterns of the broader DAA response, including DAA synthesis and turnover (racemases *alr*, *asr*, *dhaa*, *murD/F/I*, aspartate ammonia‐lyase *ansB*, and DAA dehydrogenase *dadA*).

Microbial DAA genes that showed the same pattern of differential correlation with maize genes as the pathway as a whole were distributed across the phylogeny with particularly high prevalence in alpha‐ and beta‐proteobacteria and Actinomycetes (Fig. 4c). DAA gene enrichment did not always align with a family's total functional enrichment at high temperatures. For instance, the highly abundant *Oxalobacteraceae* (likely genus *Telluria* formally *Massilia* as identified in the amplicon dataset) a core member of the maize rhizosphere microbiome associated with stress resilience (Vescio *et al*., 2021; Bowman, 2023; He *et al*., 2024). This observation, coupled with the broad distribution of DAA genes across bacterial phylogeny, indicates that this is a conserved plant‐specific mechanism of host–microbe interaction in response to abiotic stress.

The parallel changes in maize gene expression and microbial DAA metabolism suggest a synergistic relationship rather than independent responses to environmental conditions. By collapsing microbial gene expression data into pathway expression, we achieved the resolution necessary to detect these shifts which would have remained obscured by single‐gene filtering. We hypothesize that this coordination is driven by DAA signaling by the rhizosphere resulting in reactive oxygen species or other secondary signals that trigger specific physiological and chemical responses in the plant roots. While the coordinated expression patterns observed between plants and microbes across bacterial phylogeny support the existence of causal plant–microbe interactions, we acknowledge that these findings remain primarily correlative. Determining causality and the mechanisms of these exchanges will require targeted experimentation involving the use of gnotobiotic systems with known DAA‐producing isolates or the exogenous application of various DAAs to assess their direct impact on maize heat tolerance.

### Conclusion

Metatranscriptomics is a powerful alternative for microbiome profiling, revealing both the community's composition and its active functional potential, while capturing community structure similar to amplicon sequencing. Transcriptomics also enables us to treat the host and microbial transcriptomes as a single system; our approach uncovers cross‐kingdom interactions linking microbial activity to plant responses to environmental stress. The systems‐level, integrated host–microbe meta‐transcriptomic approach can be applied to many other host–microbiome systems to study the molecular basis of coordinated environmental responses.

Across all analyses, microbiome complexity, defined as the contrast between intact native field soil and depleted autoclaved soil, was the main driver of microbiome composition and function. While autoclaving provided a clear experimental contrast, we acknowledge this process alters soil physicochemical properties and selectively shifts the microbial community toward spore‐forming microbes and opportunistic colonizers from air, water, or seed sources. This resulted in a disrupted microbial landscape with significantly lower functional diversity (Fig. S1). Nevertheless, the effects of temperature and genotype, and their interactions on plant performance were observed.

Our novel integrated host–microbe metatranscriptomic analyses revealed consistent effects of temperature, genotype, and their interactions on both the plant and microbial transcriptome. By utilizing orthology‐based mapping, we identified a core set of plant genes – conserved between maize and sorghum – that respond to heat stress in tandem with microbial functional pathways. This cross‐species approach filtered out lineage‐specific functional responses, highlighting core heat stress‐mitigation strategies shared by maize and sorghum. These candidate genes are promising targets for improving plant–microbe communication and enhancing thermotolerance through breeding or biotechnology. While the scope of this study focused on conserved signatures, this dataset provides an opportunity for future studies to dissect species‐specific mechanisms of thermotolerance and microbial recruitment that reflect the distinct physiology and history of maize and sorghum.

The pathway‐level analysis of microbial transcriptomic profiles allowed us to resolve community‐level mechanisms, capturing microbiome functions that individual genes alone could not reveal. The coordinated upregulation of microbial DAA metabolism with maize genes involved in signaling, redox balance, and carbohydrate flux suggests a novel plant‐mediated synergistic feedback loop critical for genotype‐specific heat adaptation. The broad distribution of differentially correlated DAA metabolic genes across microbial phylogeny indicates that this is a conserved mechanism of host–microbe communication rather than a response limited to specific taxa. We propose that DAA turnover may act as a rhizosphere signaling cue, warranting future investigation to determine the mechanism of this molecular exchange. While DAA metabolism emerged as a key node, correlated shifts in microbial carbon and redox metabolism with plant carbohydrate and signaling genes indicate that multiple mechanisms contribute to plant/rhizosphere adaptation under heat stress.

As other studies have shown, abiotic stress causes plants to divert resources to recruit beneficial microbes; our results add to the growing body of evidence that rhizosphere microbiome regulation is an important response to abiotic stress and are among the few to generate mechanistic hypotheses (Vescio *et al*., 2021; Tiziani *et al*., 2022; Liu *et al*., 2023). To our knowledge, this is among the first studies to integrate plant and microbiome metatranscriptomes as a unified system under abiotic stress, revealing emergent patterns of functional cross‐kingdom coordination.

Collectively, these results support the GERMs framework, demonstrating that plant adaptation results from the interplay of host genetics, environmental stimuli, and microbial function. By integrating host and microbial transcriptomes, this study provides a foundation for identifying breeding targets for developing next‐generation crops capable of regulating their microbiomes to withstand a changing climate.

## Competing interests

None declared.

## Author contributions

NK, KR, MJC and JLG planned and designed the research. NK, IB and KR performed experiments and conducted glasshouse and lab work. NK and IB analyzed the data. ALW, MJC and JLG provided guidance and materials. All authors contributed to writing the manuscript.

## Disclaimer

The New Phytologist Foundation remains neutral with regard to jurisdictional claims in maps and in any institutional affiliations.

## Supporting information


**Dataset S1** Genus read counts based on 16S sequencing of the DNA.
**Dataset S2** Genus read counts based on 16S sequencing of the RNA.
**Dataset S3** Differentially expressed genes from maize and sorghum across heat and control conditions, including log‐fold changes, significance values, and orthology relationships used to compare conserved heat‐responsive pathways across species. Also includes GO terms for genes identified in intersecting tests.
**Dataset S4** List of genes differentially expressed in high temperatures in maize and sorghum from this study and 5 additional publications, and the conversion of all gene IDs to maize, B3 v5.

**Fig. S1** Microbiome complexity by soil treatment.
**Fig. S2** Additional plant phenotypes.
**Fig. S3** Proportion of reads assigned to plants and microbes.
**Fig. S4** Beta‐diversity analysis of community profiles from different sequencing types.
**Fig. S5** Comparison of sequencing methods.
**Fig. S6** Microbial functional responses to microbiome complexity, temperature, and genotype.
**Fig. S7** Random forest variable importance.
**Fig. S8** Candidate microbial mechanisms.

**Table S1** Soil chemistry profiles for field soil.
**Table S2** Full metadata for all samples, including genotype, soil treatment, temperature condition, experimental block, and phenotypic measurements used in statistical and multivariate analyses.
**Table S3** Raw sequencing read counts for plant and microbial transcriptomes.
**Table S4** Orthogroup assignments linking maize and sorghum genes, enabling cross‐species comparison of conserved genes.
**Table S5** List of microbial pathways and plant genes identified as candidates in the integrated analyses – including correlations, elastic‐net coefficients, and functional annotations – highlighting features most strongly associated with plant heat tolerance.
Please note: Wiley is not responsible for the content or functionality of any Supporting Information supplied by the authors. Any queries (other than missing material) should be directed to the *New Phytologist* Central Office.

## Data Availability

Sequenced microbiome amplicon and metatranscriptomic data are available as raw fastq files deposited in the NCBI SRA database (project accession no. PRJNA1376519). A detailed bioinformatic pipeline used in this study is available at https://github.com/NateKorth/MicrobeRNAseq.
