# Supplementary material for: Investigating GERMs: how genotype, environment, and rhizosphere microbiome interactions underlie heat response in maize and sorghum
Source: New Phytol. 2026 May 26;251(4):2152–67. doi: 10.1111/nph.71297 (PMC13373824; doi:10.1111/nph.71297)
Supplement: Supplementary file 2 — Fig. S1 Microbiome complexity by soil treatment. Fig. S2 Additional plant phenotypes. Fig. S3 Proportion of reads assigned to plants and microbes. Fig. S4 Beta‐diversity analysis of community profiles from different sequencing types. Fig. S5 Comparison of sequencing methods. Fig. S6 Microbial functional responses to microbiome complexity, temperature, and genotype. Fig. S7 Random forest variable importance. Fig. S8 Candidate microbial mechanisms. [file NPH-251-2152-s001.pdf]

New Phytologist Supporting Information

Article title: **Investigating GERMs: How Genotype, Environment, and Rhizosphere Microbiome interactions underlie heat response in maize and sorghum**

Authors: Nate Korth*, Isabella Borrero, Katelyn Rumley, Alex L. Woodley, Mallory J. Choudoir, Joseph L. Gage

*Corresponding Author: njkorth@ncsu.edu

Article acceptance date: 8 May 2026

Supplemental Figure 1

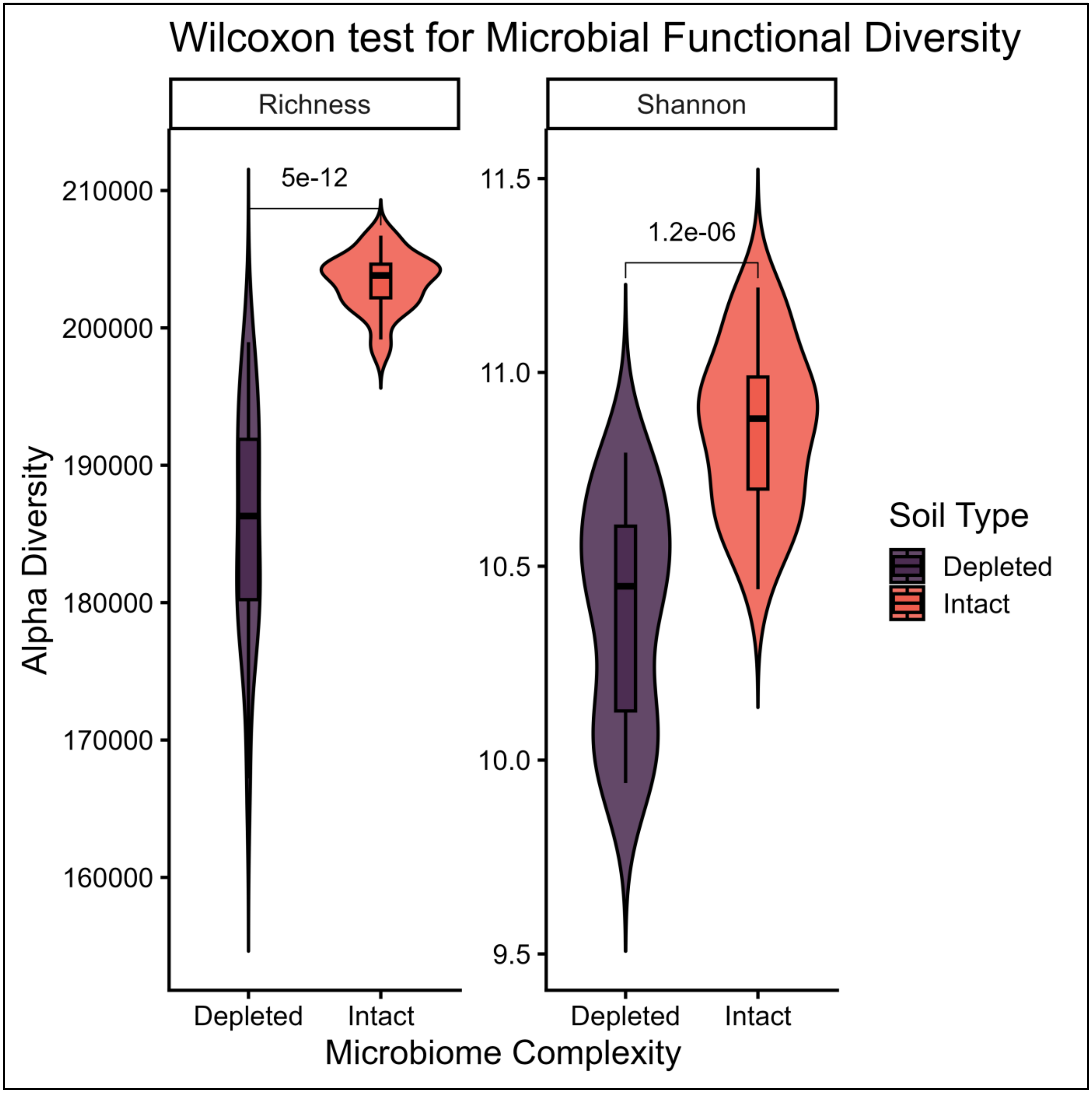

Supplemental Figure 1. Microbiome complexity by soil treatment. *The diversity in the transcriptome shown as the total number of unique transcripts (Richness) and Shannon diversity between all samples. These metrics demonstrate the autoclaving process effectively depleted microbial functionality with significantly lower functional diversity compared to the intact field soil (Wilcoxen test; p < 0.05).*

Supplemental Figure 2

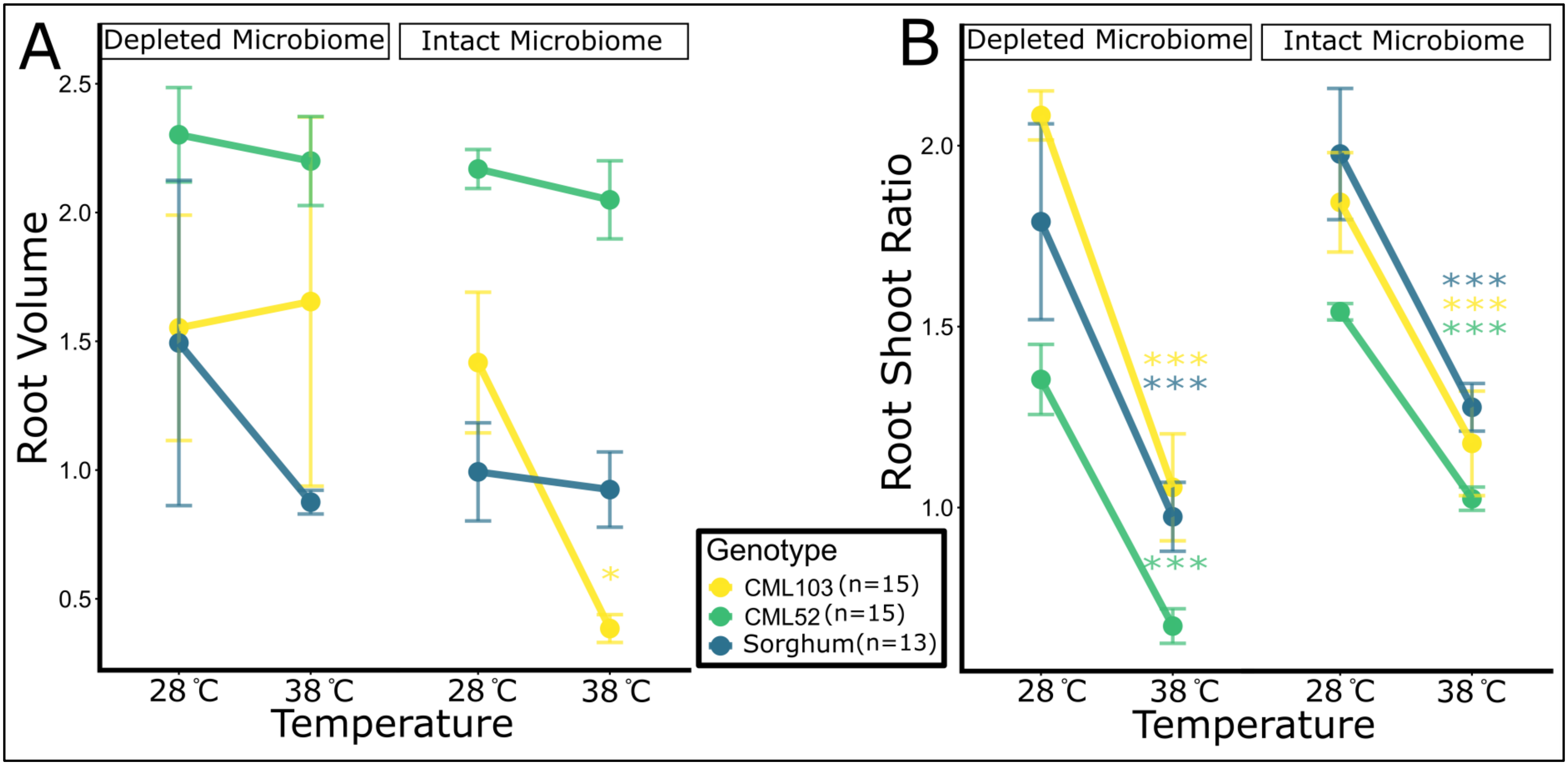

Supplemental Figure 2. Additional plant phenotypes. *Root volume was relatively stable in heat-tolerant plants and decreased rapidly in susceptible genotypes under heat stress (A). Temperature had a consistent effect on the root-to-shoot ratio across levels of microbiome complexity (B). Asterisks denote significant differences in temperatures within each microbiome-genotype combination, as determined by Tukey's HSD (* $p<0.05$, ** $p<0.01$, *** $p<0.001$).*

Supplemental Figure 3

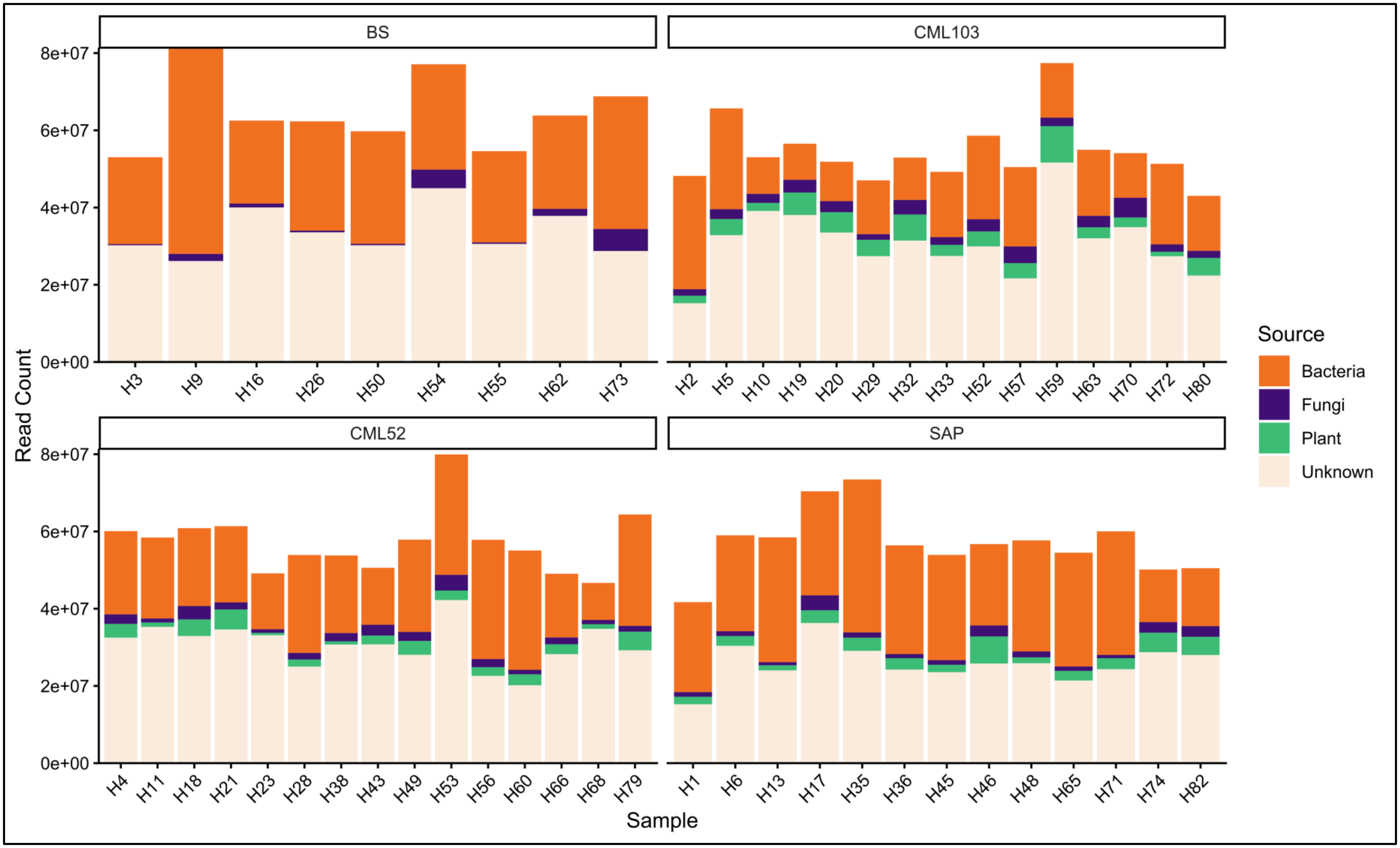

Supplemental Figure 3. Proportion of reads assigned to plants and microbes. *Read counts of all samples faceted by genotype and colored by assignment to plant, bacteria, or fungi. BS refers to bulk soil samples.*

Supplemental Figure 4

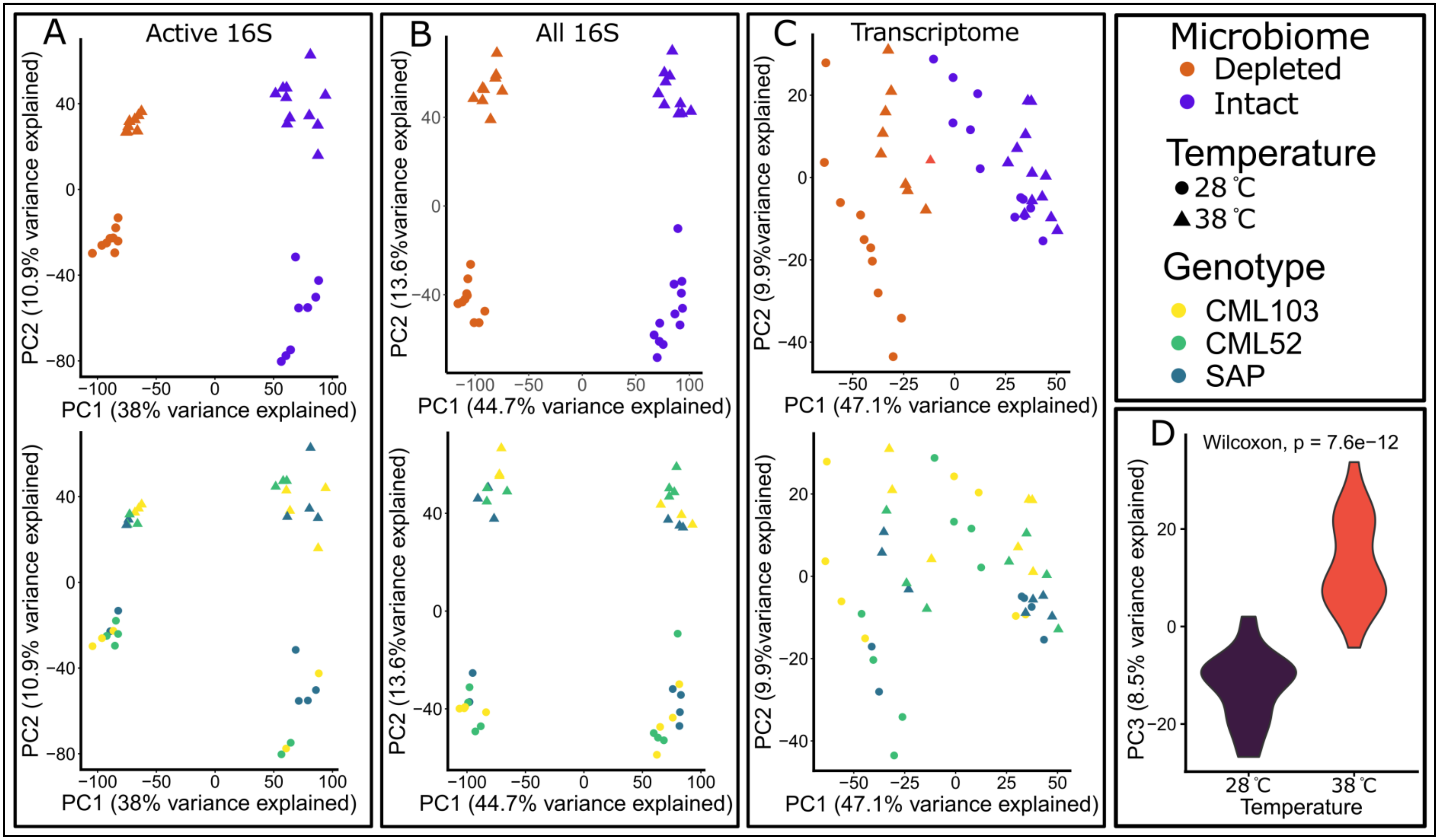

Supplemental Figure 4. Beta-diversity analysis of community profiles from different sequencing types. *Beta-diversity (Aitchison's PCoA) of data from active 16S (A), all 16S (B), and transcriptomics (C). Points are colored by microbiome complexity (top) and plant genotype (bottom) and shaped by Temperature. PC3 is shown for transcriptomics, as it resolves differences in temperature (D).*

Supplemental Figure 5

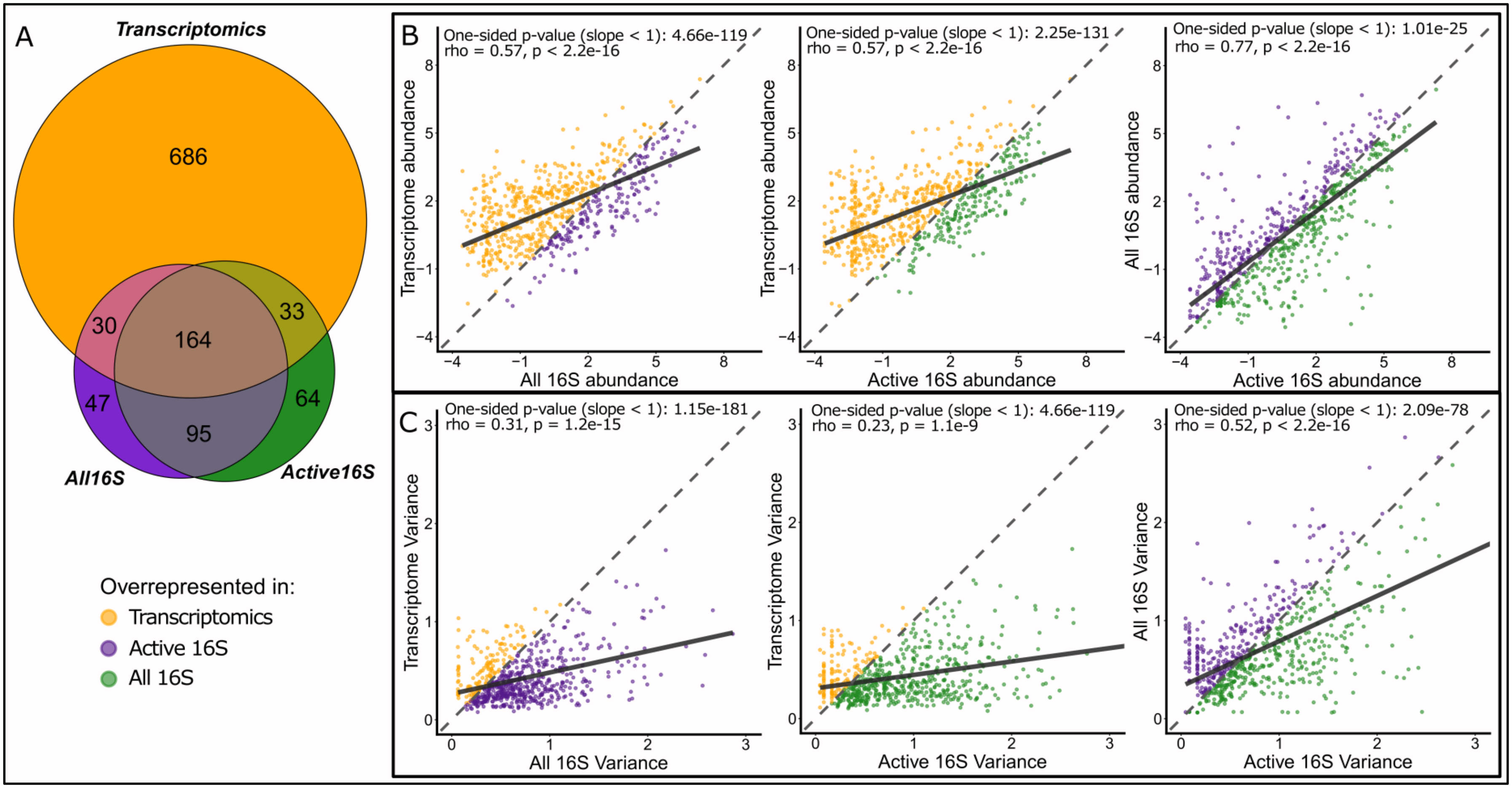

Supplemental Figure 5. Comparison of sequencing methods. *Venn diagram showing shared bacterial genera among sequencing types. Metatranscriptomics (orange) captured more unique genera than either all 16S (green) or active 16S (purple) (A). Correlation of abundance between sequencing methods, showing enhanced detection of low-abundance taxa in transcriptomics (B). Lower variation between replicates in transcriptomics indicates greater reproducibility (C). In all dot plots, the Spearman correlation trend lines are shown in black, and points are colored based on deviation from the expected 1:1 slope.*

Supplemental Figure 6

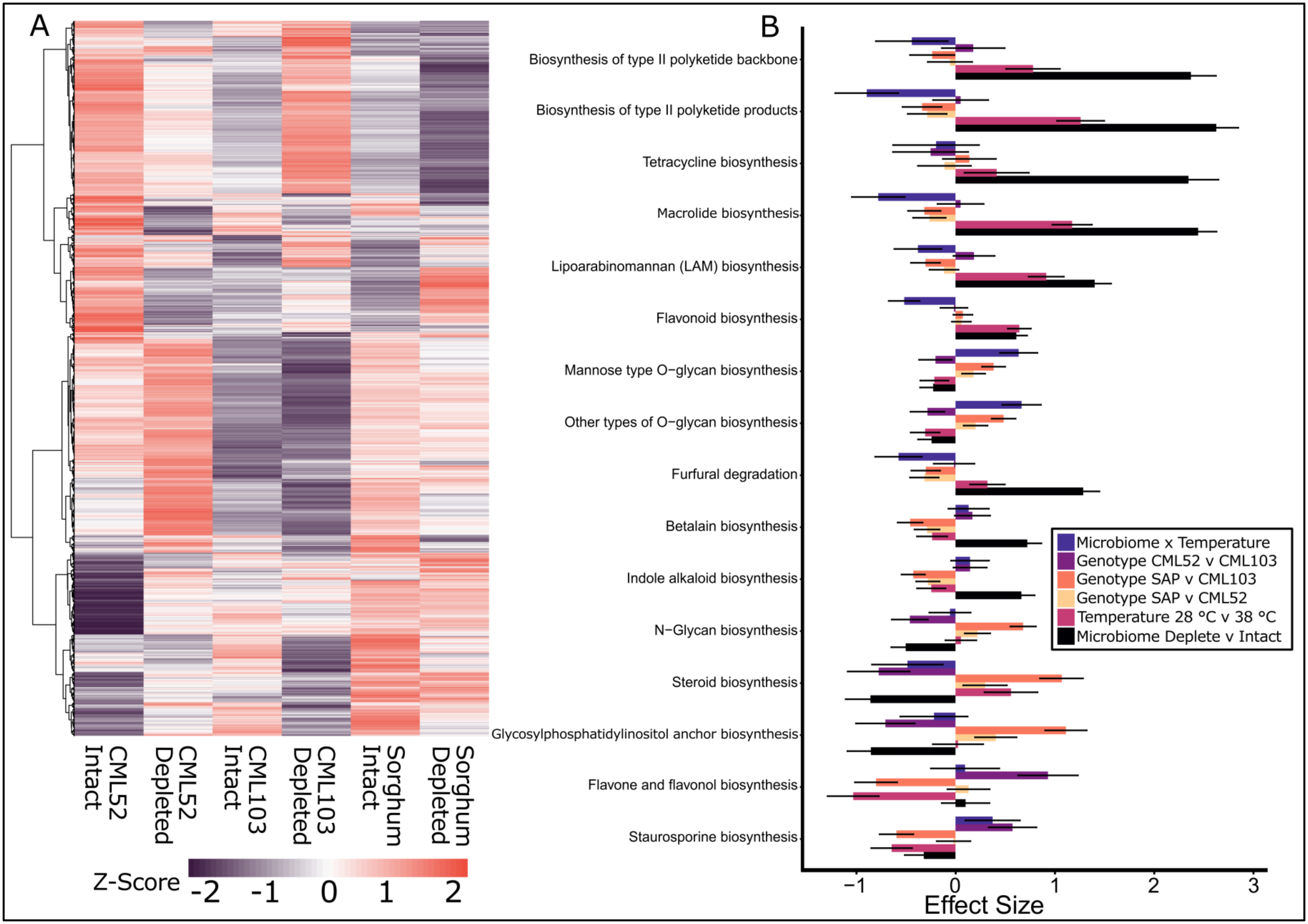

Supplemental Figure 6. Microbial functional responses to microbiome complexity, temperature, and genotype. *Heatmap of normalized expression (Z-score) for differentially expressed (temperature) microbial genes across samples. Hierarchical clustering was performed on genes (rows) using Euclidean distance. Red indicates higher relative expression, and purple indicates lower relative expression. Sample labels indicate genotype and microbiome complexity(A). Effect sizes for the top microbial pathways for main and interaction effects, derived from the ALDEx2 GLM. Bars represent estimated effect sizes for the microbiome, temperature, genotype, and the microbiome-temperature interaction. Bars represent standard errors (B).*

Supplemental Figure 7

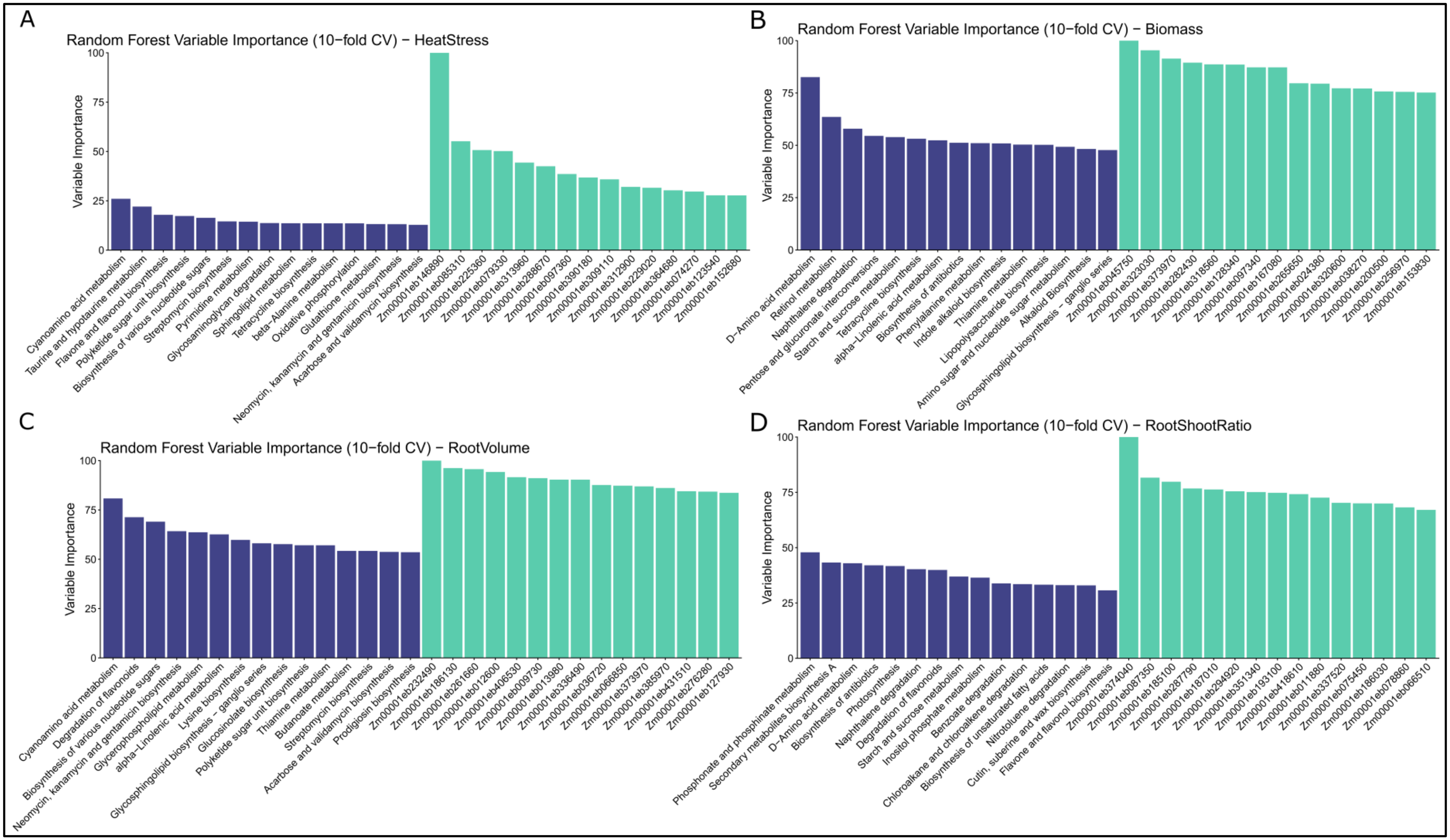

Supplemental Figure 7. Random forest variable importance. *The top 15 microbial pathways and maize genes identified by a random forest model for predicting biomass using 2,000 trees and 10-fold cross-validation for heat stress (A), biomass (B), root volume (C), and root-to-shoot ratio.*

Supplemental Figure 8

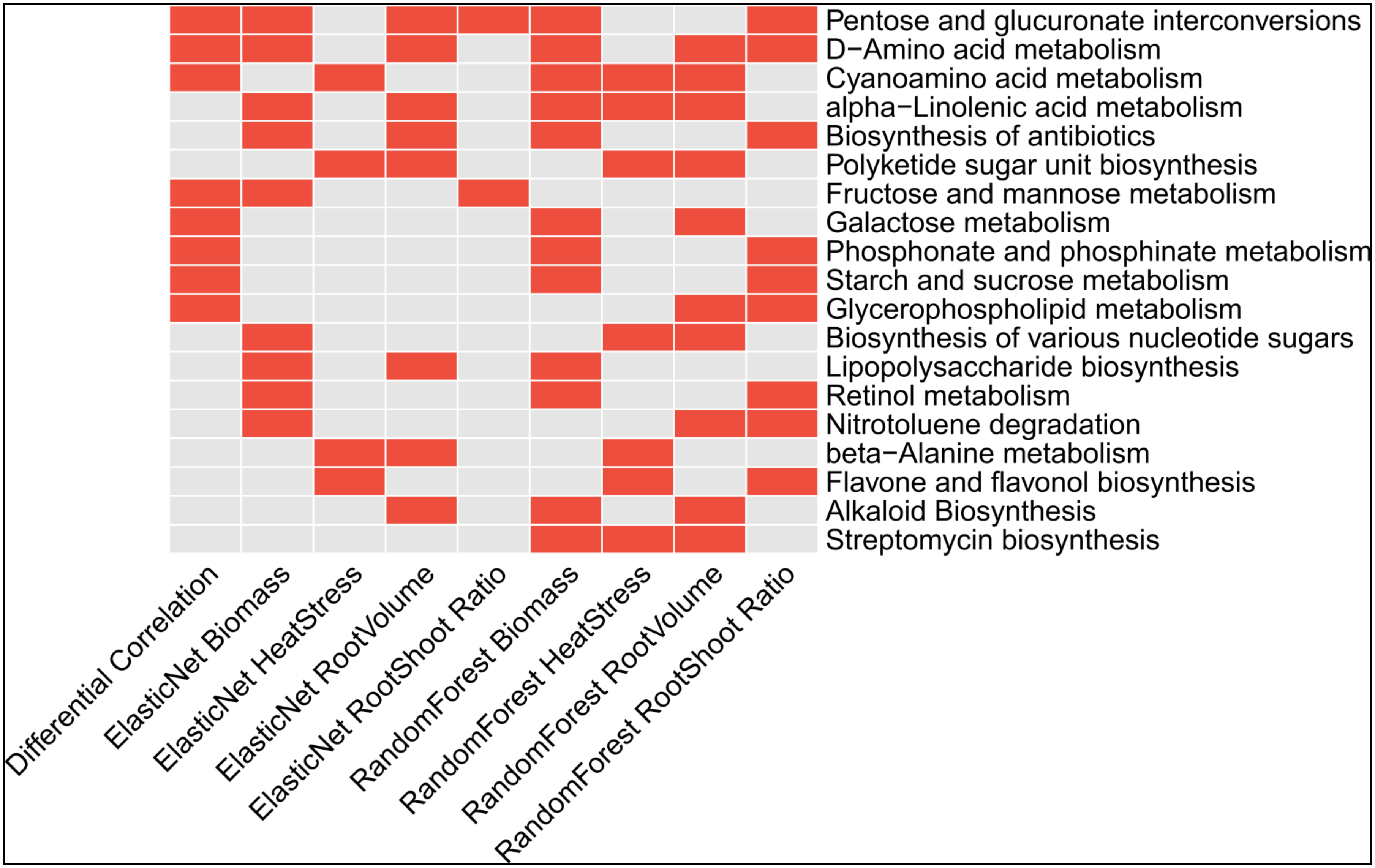

Supplemental Figure 8. Candidate microbial mechanisms. *Microbial Pathways present in at least three analyses of the differential bipartite correlation, Elastic Net, or the Random Forest, using biomass, heat stress, or root volume as the response variable.*
